# A performance evaluation of chemiluminescence enzyme immunoassays on the Sysmex CN‐6500 haemostasis analyser

**DOI:** 10.1111/ijlh.13656

**Published:** 2021-07-12

**Authors:** Chris Gardiner, Philip Lane, Hitesh Tailor, Samuel J. Machin, Ian J. Mackie

**Affiliations:** ^1^ Research Department of Haematology University College London London UK; ^2^ Chris Gardiner Consulting Ltd. Aylesbury Buckinghamshire UK; ^3^ Haematology Evaluations Unit HSL (Analytics) LLP London UK

**Keywords:** coagulation, DIC, fibrinolysis, laboratory automation

## Abstract

**Background:**

The Sysmex CN‐6500 is a new haemostasis analyser with an integrated immunoassay module that performs chemiluminescence enzyme assay (CLEIA) in addition to coagulation, turbidimetric, chromogenic and platelet aggregation tests.

**Aims:**

To evaluate the analytical performance of the CN‐6500 against the predicate device (Sysmex HISCL‐800) for soluble thrombomodulin (TM), thrombin‐antithrombin (TAT), tissue plasminogen activator/plasminogen activator inhibitor 1 complex (tPAI‐C) and plasmin α2 plasmin inhibitor complex (PIC) assays.

**Methods:**

Imprecision was assessed by testing two levels of quality control plasmas 10 times on 5 separate days. Comparability was studied in 230 plasmas from normal donors (n = 30), patients with suspected disseminated intravascular coagulation (DIC, n = 100), sepsis (n = 20) or liver disease (n = 20), lipaemic (n = 20), haemolysed (n = 20) and icteric samples (n = 20). Limit of detection, limit of quantitation and linearity were determined by testing serial dilutions of normal plasma. Sample carryover was assessed by testing samples with high and low normal levels of the analytes concerned.

**Results:**

The CN‐6500 performed 21 CLEIA tests per hour, while simultaneously performing coagulation tests. Acceptable between‐run imprecision was obtained using commercial controls with normal and high activity for each analyte (%CV <4%), for all four assays. Excellent linearity was observed (slope 0.89‐1.03; r^2^ >0.99) across the measurement range. The lower limits of detection and quantitation were as follows: TM <0.3/0.6 TU/ml, TAT >0.1/<0.2 ng/ml, PIC <0.004/<0.008 µg/ml and tPAI‐C < 0.01/<0.1 ng/ml, respectively. All four assays showed excellent correlation between analysers and were unaffected by haemolysis, icterus or lipaemia. No carryover was observed.

**Conclusions:**

Our data demonstrate that the performance of the CLEIA assays on the CN‐6500 is comparable to that of a stand‐alone immunoassay analyser.

## 1 INTRODUCTION

The Sysmex CN‐series (Sysmex Corporation) is a range of high‐throughput compact haemostasis analysers.^1^ The CN‐6500 analyser has an integral immunoassay module which performs chemiluminescence enzyme immunoassays (CLEIA) in addition to coagulation, chromogenic, immunoturbidometric and light transmission platelet aggregometry testing. CLEIA is an immunoassay technique in which the label is a luminescent molecule. It typically offers a wide linear measurement range, with a high signal intensity, and an absence of interference, resulting in high sensitivity and specificity.^2^ In common with other automated CLEIA systems, the CS‐6500 is much faster than traditional immunoassays.

The CN‐6500 currently has four CLEIA assays available; soluble thrombomodulin (TM), thrombin‐antithrombin (TAT), tissue plasminogen activator/plasminogen activator inhibitor 1 complex (tPAI‐C) and plasmin/α2 plasmin inhibitor complex (PIC). The performance of these assays has previously been evaluated on the HISCL‐5000 (Sysmex Corporation),^3^ a stand‐alone fully automated rapid immunochemistry system which utilizes CLEIA technology. The HISCL system uses biotinylated primary antibodies, streptavidin‐coated magnetic beads, alkaline phosphatase (ALP) labelled secondary antibodies and an ALP chemiluminescent substrate (CDP‐Star®). These assays are reported to have clinical utility in the diagnosis of disseminated intravascular coagulation (DIC) and its subtypes.^3^, ^4^, ^5^ They also have potential applications in cancer medicine.^6^ The diagnostic criteria for DIC proposed by the Japanese Society for Thrombosis and Haemostasis recognize different subtypes of DIC, which reflect fibrinolytic status.^4^ Consequently, the measurement of TAT and PIC is frequently included in the diagnostic workup for DIC in Japanese laboratories. Endogenous soluble TM may be assayed as a marker of endothelial injury, as it is cleaved and released from the vessel wall in many inflammatory conditions.^7^ tPAI‐C may also be a useful assay in the investigation of DIC, and increased levels are associated with multiorgan failure.^8^ More recently, it has been observed that TM, TAT, PIC and tPAI‐C may have prognostic value in assessing patients with cancer^6^ COVID‐19.^9^, ^10^

The purpose of this study was to compare the performance of four CLEIA tests on the CN‐6500 haemostasis analyser against the same reagents on the predicate device (HISCL‐800).

## 2 METHODS

The analytical performance of the CN‐CLEIA system was compared with that of a stand‐alone immunoanalyser (Sysmex HISCL‐800) for TM, TAT, PIC and tPAI‐C.^6^, ^7^ The CN‐6500 performed the CLEIA method as follows: 10‐30μl of sample was incubated with a primary biotinylated antibody in the liquid phase to allow antibody‐antigen binding. Streptavidin‐coated magnetic beads were added to bind antibodies, and free antigen was removed by washing. Following a brief incubation with an ALP labelled secondary, unbound secondary antibody is removed by washing. Buffer was added to disperse the beads, the ALP chemiluminescent substrate (CDP‐Star®) was added and luminescence intensity measured. TAT and tPAI‐C were reported in ng/ml, TM was reported in TU/ml (thrombomodulin units), and PIC was reported in µg/ml, as per the manufacturer's instructions. Precision was assessed using Controls L and H (normal and high QC materials) provided by the manufacturer tested 10 times on each of 5 separate days.

Citrated plasma was obtained from residual, anonymized samples (collected into 0.109 mol/L sodium citrate (Vacutainer; Becton Dickinson) after all routine testing had been completed, in compliance with local ethical committee rules and the Human Tissues Act 2004 (UK). Samples were frozen at −8°C and thawed in a 37°C water bath immediately prior to testing. Normal citrated plasma collected locally from apparently normal healthy volunteers and commercially sourced plasmas (CRYOcheck™ Normal Donor Set; Precision BioLogic Inc) were also tested. Informed consent was obtained from normal donors (approved by the UCL Research Ethics committee: Project ID Number: 7029/001).

Linearity, lower limit of detection (LLoD) and lower limit of quantitation (LLoQ) were determined by serial dilution of pathological plasma samples in assay diluent across the reportable range. For LLoD and LLoQ determination, each dilution was tested six times. The LLoD was the lowest concentration showing a statistically significant difference from the assay blank. This was achieved by determining the mean concentration at each dilution and performing a T test relative to six blank measurements. The LLoQ was the lowest concentration which gave a %CV of <10%).

Sample carryover was investigated as previously described.^11^ Briefly, aliquots of normal plasma (A1‐A3) and plasma containing high levels of the analyte of interest (B1‐B3) were tested in the sequence A1, A2, A3, B1, B2, B3 five times, then repeated with the sequence in reverse.

Unfractionated heparin (5000 IU/ml Multiparin, Wockhardt UK Ltd) was added to samples at 2.0 U/ml and compared with the unadulterated samples to study possible interference.

Comparability was assessed in 230 plasmas from normal donors (n = 30), patients with suspected DIC (n = 100), sepsis (n = 20), liver disease (n = 20), lipaemia (n = 20), haemolysis (n = 20) and icterus (n = 20). The samples from patients suspected DIC were from the intensive therapy unit (ITU) with low platelet counts. Samples with haemolysis, icterus or lipaemia were selected by visual inspection. Plasma haemoglobin was assayed using the HemoCue system (Radiometer). Cholesterol and triglycerides were measured using Chol2 and triglyceride reagents using a Cobas C 111 analyser (Roche Diagnostics).

Throughput was assessed by three different exercises. All throughput exercises were started from when the instrument was at rest, in reflex testing mode.

- measurement of TM, TAT, PIC and tPAI‐C alone in 12 samples from patients on ITU
- simultaneous measurement of the four CLEIA methods, plus prothrombin time (PT), activated partial thromboplastin time (APTT), Clauss fibrinogen assay (Fib) and D‐dimer (Innovin^®^, Actin^®^ FS, Thrombin Reagent and INNOVANCE^®^ D‐dimer, all Siemens, Marburg, Germany) in 12 samples from patients on ITU
- measurement of PT, APTT, Fib and D‐dimer only on 12 samples from patients on ITU
- PT, APTT, Fib and D‐dimer in 100 samples selected at random from the clinical workload.

Statistical analysis was performed with GraphPad Prism version 9.0.0 for Windows (GraphPad Software). Reference intervals were calculated as the 2.5th‐97.5th percentiles after determining that the data had a non‐normal distribution. Comparability was assessed by Deming regression analysis, and non‐parametric methods were used throughout. Probability values (*P*) <.05 were considered statistically significant. Systemic bias was obtained by Bland‐Altman analysis.

## 3 RESULTS

Ten replicate tests of high and low QC preparations tested over 5 days gave coefficients of variation of <4.0% for both within‐ and between‐run imprecision (Table 1) for all four assays. The target values for the QC materials were assigned for the HISCL‐800 only, so these values were used for both instruments. It was not always possible to use the same QC lots on both instruments. Some of the mean values, particularly for TM and PIC, showed bias of up to 25% relative to manufacturer's target values for both the CN‐6500 and the HISCL‐800. The manufacturer's reference (RI) was verified by testing 30 samples from normal donors by all four assays on both instruments, with ≥90% of values lying within the stated RI^12^ with all methods, except PIC on the CN‐6500 (Table 2).

**TABLE 1:** Between‐run imprecision for low and high QC preparations

	HISCL‐800 QC H					CN‐6500 QC H				
Assay	Target	Mean	Bias	SD	%CV	Target	Mean	Bias	SD	%CV
TM (TU/ml)	91.8	113.0	23.1%	2.5	2.17%	91.8	115.0	25.3%	3.8	3.29%
TAT (ng/ml)	39.1	36.9	−5.6%	1.4	3.77%	38.1	33.6	−11.8%	0.9	2.78%
PIC (μg/ml)	7.217	7.257	0.6%	0.254	3.49%	8.007	7.160	−10.6%	0.193	2.70%
tPAI‐C (ng/ml)	17.6	15.6	−11.4%	0.62	3.98%	17.5	17.46	−0.2%	0.50	2.85%
	HISCL‐800 QC L					CN‐6500 QC L				
Assay	Target	Mean	Bias	SD	%CV	Target	Mean	Bias	SD	%CV
TM (TU/ml)	22.6	27.4	21.2%	0.7	2.48%	22.6	27.7	22.6%	0.8	2.93%
TAT (ng/ml)	10.0	9.6	9.6%	0.3	3.36%	9.5	8.5	−10.5%	0.2	2.27%
PIC (μg/ml)	2.071	1.773	−14.4%	0.056	3.22%	2.071	1.620	−21.8%	0.046	2.84%
tPAI‐C (ng/ml)	4.6	4.1	−10.0%	0.15	3.75%	4.7	4.6	−2.6%	0.13	2.79%

Note

10 replications on each of 5 different days. The mean bias of measured values against the manufacturer's assigned target value for HISCL‐800 are also shown.

**TABLE 2:** Reference interval verification in 30 samples from normal donors (2.5th–97.5th percentiles)

Assay	Manufacturer's reference interval (HISCL‐800)	HISCL‐800 (n = 30)	CN‐6500 (n = 30)
TM	3.8‐13.3 TU/ml	6.3‐11.3	6.9‐14.5
TAT	0.6‐3.9ng/ml	0.40‐3.12	0.40‐3.25
PIC	0.24‐0.89㎍/ml	0.254‐1.258	0.293‐1.319
tPAI‐C	3.76‐14.0ng/ml	1.82‐13.46	2.23‐18.72

Note

3, 5 and 3 samples gave values above the manufacturer's RI for TM, PIC and tPAI‐C using the CN‐6500. 3 samples produced a PIC value above the manufacturer's RI using the HISCL‐800. 4 samples gave TAT gave values below the manufacturer's RI with both analysers. The HISCL‐800 and CN‐6500 produced tPAI‐C values below the manufacturer's RI in 11 and 7 samples, respectively.

**FIGURE 1:**
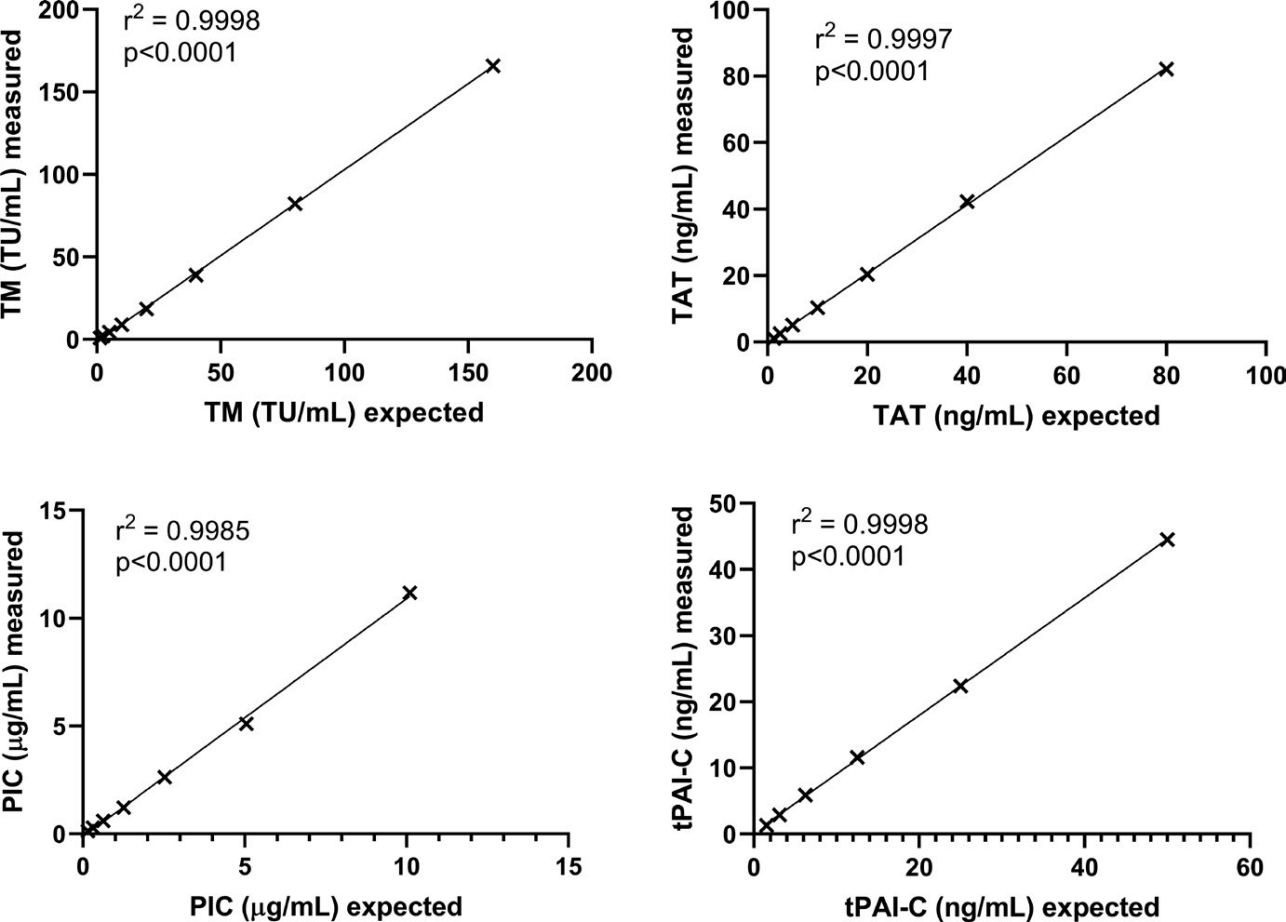
Linearity for TM, TAT, PIC and tPAI‐C assessed in doubling dilutions of plasma

To assess linearity, doubling dilutions of four plasma samples with high levels of TM. TAT, PIC and tPAI‐C, as determined by the predicate method, were made manually in assay diluent. The measured concentrations were plotted against the expected, calculated concentration. Excellent linearity was observed for all four assays across the reportable range, with r^2^ values of 0.998 or better (Figure 1). The lower limit of quantitation (LLoQ) for TM, TAT, PIC and tPAI‐C was 0.6 TU/ml, 0.2 ng/ml, 0.008 μg/ml and 0.1 ng/ml, respectively. Lower limit of detection (LLoD) was 0.3 TU/ml, 0.1 ng/ml, 0.004 μg/ml and 0.01 ng/ml.

Optical interference was assessed in 60 samples with lipaemia, icterus or haemolysis with triglycerides up to 26.1 mmol/L, cholesterol up to 10.3 mmol/L, bilirubin up to 531 μmol/L and haemoglobin up to 15.5 g/L. No optical interference was evident, with good agreement between the two methods, and results were obtained for TM, TAT PIC, and tPAI‐C in all 60 samples. Activation in two haemolysed samples caused unstable levels of TAT, and these samples were excluded from the analysis. In vitro haemolysis is known to be associated with activation of the coagulation pathways.^13^ No significant sample carryover was detected in the TAT and tPAI‐C assays; TAT normal to abnormal carryover 2.16%, abnormal to normal 0.00%, tPAI‐C normal to abnormal carryover 1.4%, abnormal to normal −0.07%. Carryover testing was not performed on the TM and PIC assays. Unfractionated heparin added to samples at a level of 2.0 IU/ml had no demonstrable effect on the CLEIA assays, with differences of 2.07%, 0.00%, 1.30% and 0.88% for TM, TAT, PIC and tPAI‐C, none of which were statistically significant.

**FIGURE 2:**
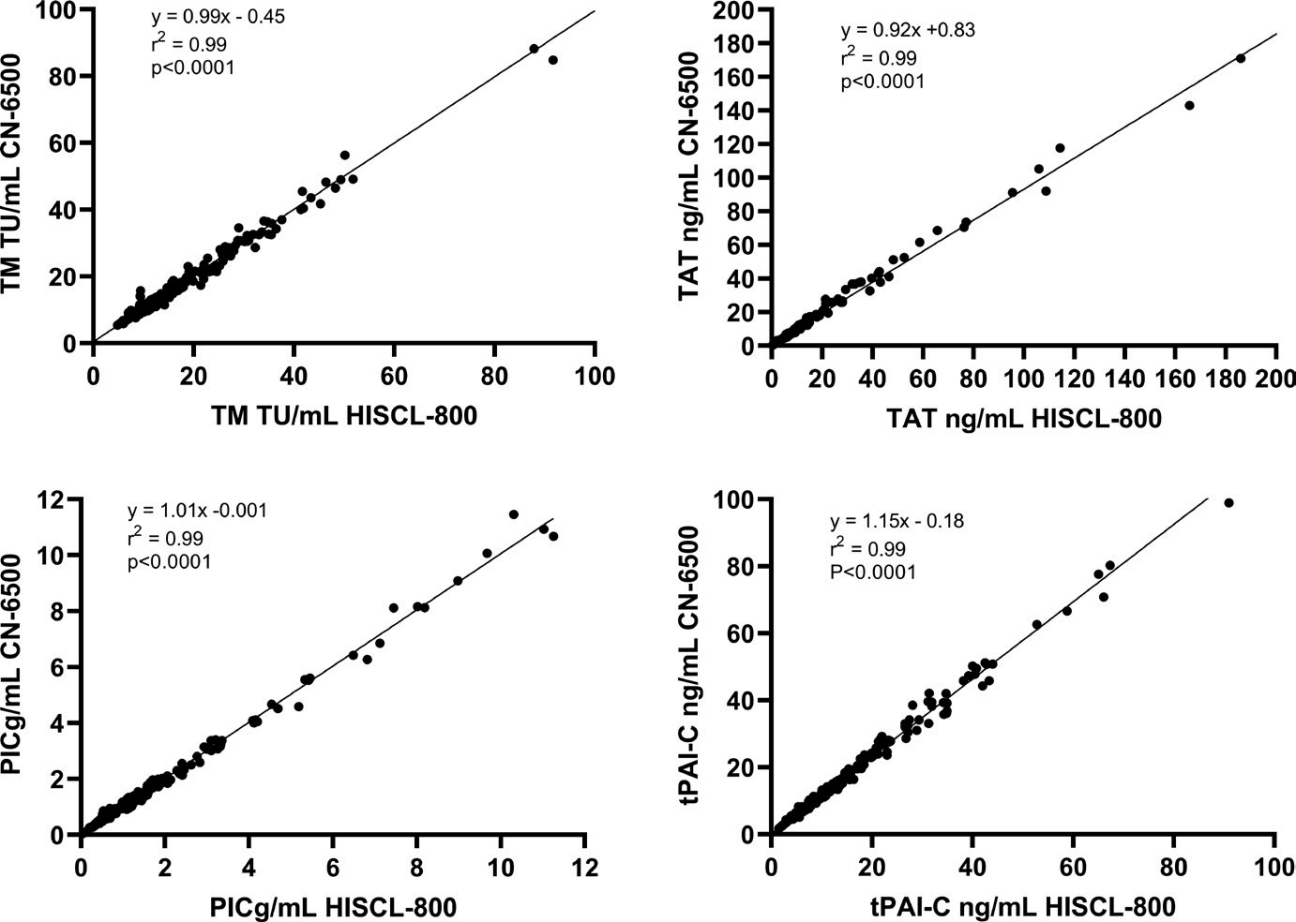
Comparability of TM, TAT, PIC and tPAI‐C in 230 clinical plasma samples. The 95% CI for the slopes were TM 0.947 to 1.036, TAT 0.875 to 0.971, PIC 0.970 to 1.040 and tPAI‐C 1.117 to 1.189, The 95% CI for the y‐intercepts were TM −0.238 to1.131, TAT −0.381 to 1.283, PIC −0.0455 to 0.0155 and tPAI‐C −0.241 to 0.598. The p value indicates the significance of the slope of the linear regression from zero. The p value indicates the significance of the slope of the linear regression from zero

**FIGURE 3:**
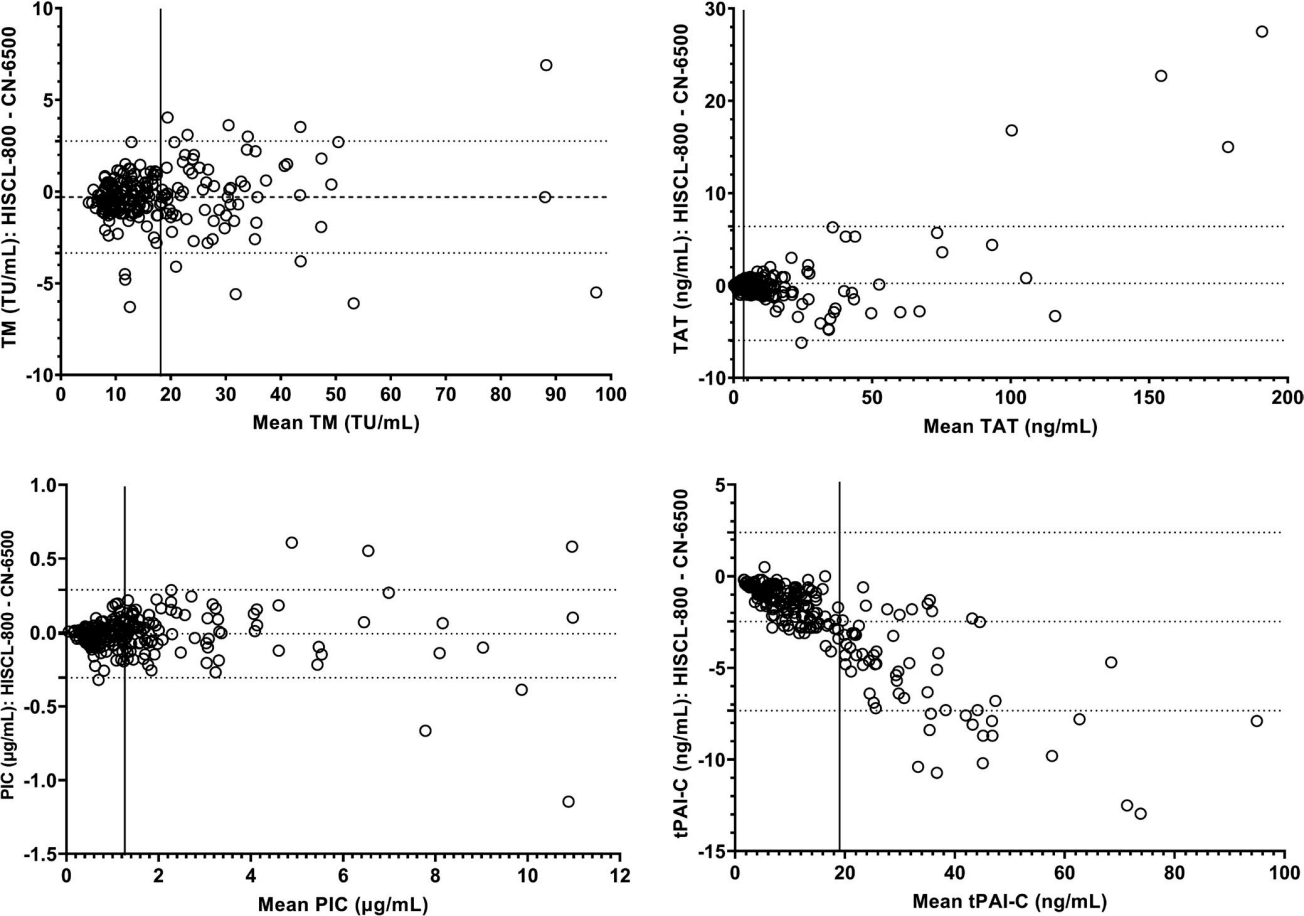
Bland‐Altman analysis of TM, TAT, PIC and tPAI‐C in 230 clinical samples. The bias and 95% limits of agreement were TM −0.3 TU/ml (−3.4 to 2.8 TU/ml), TAT 0.2 ng/ml(−5.9 to 6.4 ng/ml), PIC −0.009 µg/ml (−0.307 to 0.289 µg/ml) and tPAI‐C −2.47 ng/ml (−7.34 to 2.39 ng/ml). The solid vertical lines represent the upper limit of the reference interval

The two instruments demonstrated excellent correlation across the reportable range in 230 samples tested (Figure 2). The CN‐6500 displayed the following systemic bias (calculated from the Bland Altman analysis), relative to the predicate device; TM −0.3 TU/ml, TAT 0.2 mg/ml, PIC −0.009 μg/ml (0.1%) and tPAI‐C −2.5 ng/ml (Figure 3). tPAI‐C showed increasing proportional bias with rising concentration. TAT also demonstrated a small but statistically significant proportional bias. Three plasmas with tPAI‐C results above the reportable range by both methods were excluded from the analysis.

The CLEIA throughput was 21 tests per hour (12 samples tested by all 4 methods in 2 hours 15 minutes), with the first result available in 17 minutes. The CLEIA throughput rate was not significantly affected by simultaneously running coagulation tests. The time taken to analyse PT, APTT, Fib and D‐dimer while simultaneously performing the four CLEIA tests on the same samples was 23 minutes and 30 seconds, while the time taken for PT, APTT, Fib and D‐dimer alone was 19 minutes 35 seconds. The throughput for PT, APTT, Fib and D‐dimer was 369 tests per hour. These times are inclusive of reflex testing for samples with high Fib and/or D‐dimer results.

## 4 DISCUSSION AND CONCLUSIONS

The CN‐6500 is the first routine haemostasis analyser to incorporate a chemiluminescence module. At present, only TM, TAT, PIC and tPAI‐C assays are available on the CN‐6500 but it is expected that other assays will be added following this successful proof of concept. At present, twenty immunoassays are available on the HISCL‐800 for a wide range of analytes associated with coagulation^5^, ^14^ (eg PIVKA), tumour markers, virology and immunology.^15^, ^16^ During this performance evaluation, we verified that the CN‐6500 can perform coagulation tests and CLEIA tests simultaneously, with no drop in throughput (CLEIA throughput 21 tests per hour). When performing coagulation tests simultaneously with CLEIA, the CLEIA throughput is not significantly altered but coagulation test throughput is slightly reduced, due to increased sampling time. The manufacturer states that the onboard stability of the reagents is 30 days, but that was not verified during this evaluation. Although the level of imprecision obtained by repeated testing of high and low QC materials was acceptable, significant bias was observed between results obtained with both analysers and the manufacturer's assigned target values for the HISCL‐800. Specific target values for the CN‐6500 were not available. We have raised our concerns regarding the QC target values with the manufacturer.

Excellent linearity was obtained in a range of dilutions covering the reportable range. LLoD and LLoQ were within the manufacturer's specifications for all four assays. All were significantly lower the bottom of the reference interval. No carryover issues were identified, and no interference due to heparin, lipaemia, icterus or haemolysis was observed. The multiple washing processes used the CN‐6500, coupled with chemiluminescent detection should eliminate optical interference. We felt that comparison to the predicate method was sufficient in this instance, as it has previously been shown to be unaffected by optical interference.^3^ The manufacturer's reference intervals were verified for TM, TAT, and tPAI‐C, but we obtained a higher RI for PIC with both analysers. This may be due to the ethnicity of the normal donors (the manufacturer's RI was determined in Japanese donors).^17^ We would recommend establishment of a local RI in at least 120 normal healthy individuals representative of the local population prior to introducing these assays into clinical use. The correlation between the HISCL‐800 and CN‐6500 was excellent in a wide range of clinical samples covering the reportable range for all four CLEIA parameters and gave results that were comparable to a previous study.^5^ However, the CN‐6500 produced t‐PAIC values with a significant bias, which indicates a problem with the calibration of this assay. However, agreement within the reference interval was acceptable (Figure 3), so the differences at higher concentrations are unlikely to be of clinical significance. Nevertheless, we have raised concerns regarding possible calibration issues with the manufacturer.

We conclude that the performance of the CLEIA assays on the CN‐6500 is comparable to that of a stand‐alone immunoassay analyser.

## CONFLICTS OF INTEREST

This work was supported by an institutional, unrestricted research grant from Sysmex UK. CG, IJM and SJM are consultants for Sysmex Corp.

## Data Availability

The data that support the findings of this study are available from the corresponding author upon reasonable request.
